# Validation of a novel automatic deposition of bacteria and yeasts on MALDI target for MALDI-TOF MS-based identification using MALDI Colonyst robot

**DOI:** 10.1371/journal.pone.0190038

**Published:** 2017-12-29

**Authors:** Katerina Chudejova, Michal Bohac, Anna Skalova, Veronika Rotova, Costas C. Papagiannitsis, Jana Hanzlickova, Tamara Bergerova, Jaroslav Hrabák

**Affiliations:** 1 Biomedical Center and Department of Microbiology, Faculty of Medicine and University Hospital in Pilsen, Charles University, Plzen, Czech Republic; 2 Bruker s.r.o., Brno, Czech Republic; 3 Department of Microbiology, University Hospital in Pilsen, Plzen, Czech Republic; Universita degli Studi di Parma, ITALY

## Abstract

Matrix-assisted laser desorption/ionization time-of-flight mass spectrometry (MALDI-TOF MS) -based identification of bacteria and fungi significantly changed the diagnostic process in clinical microbiology. We describe here a novel technique for bacterial and yeast deposition on MALDI target using an automated workflow resulting in an increase of the microbes’ score of MALDI identification. We also provide a comparison of four different sample preparation methods. In the first step of the study, 100 Gram-negative bacteria, 100 Gram-positive bacteria, 20 anaerobic bacteria and 20 yeasts were spotted on the MALDI target using manual deposition, semi-extraction, wet deposition onto 70% formic acid and by automatic deposition using MALDI Colonyst. The lowest scores were obtained by manual toothpick spotting which significantly differ from other methods. Identification score of semi-extraction, wet deposition and automatic wet deposition did not significantly differ using calculated relative standard deviation (RSD). Nevertheless, the best results with low error rate have been observed using MALDI Colonyst robot. The second step of validation included processing of 542 clinical isolates in routine microbiological laboratory by a toothpick direct spotting, on-plate formic acid extraction (for yeasts) and automatic deposition using MALDI Colonyst. Validation in routine laboratory process showed significantly higher identification scores obtained using automated process compared with standard manual deposition in all tested microbial groups (Gram-positive, Gram-negative, anaerobes, and yeasts). As shown by our data, automatic colony deposition on MALDI target results in an increase of MALDI-TOF MS identification scores and reproducibility.

## Introduction

Fully automated clinical microbiology laboratory is currently a high priority development project of several commercial companies [1]. In contrast to biochemical or hematology laboratories, which use standard tubes and have minimal diversity of materials, microbiology must accept plenty of sorts of specimen or transport media. Therefore, there is a problem of standardization of diagnostic processes. The situation is, however, improving using new technologies such as mass spectrophotometry, molecular techniques or automatic lines [2].

Automated process is expected to positively affect the quality of processed specimens and their standardization, including documentation of microbial cultures on plates. Automatic lines currently include automation of sample inoculation process, smart incubation with possible documentation of microbial growth on the plates and workbenches allowing plate reading via high-resolution imaging [1,3].

Most important aspect in clinical microbiology is a taxonomical identification of cultivated microbes. In the past, identification of bacteria and yeasts, was based mainly on biochemical detection of enzymes produced by microorganisms. In the last decade, introduction of matrix-assisted laser desorption ionization-time of flight mass spectrometry (MALDI-TOF MS) revolutionary changed identification process by its accuracy and rapidness [4,5,6].

MALDI-TOF MS identification increased workflow efficiency having turnaround time of 6–10 minutes per identification of one isolate and reduced the cost per identification [3]. This technique allows precise identification of most microbes comparing with biochemical tests. Only some species (e.g., *Streptococcus* spp., *Enterobacter* spp., some Gram-negative non-fermenting rods) are indistinguishable by MALDI-TOF MS identification [6]. *Mycobacterium* spp., *Nocardia* spp., *Actinomyces* spp. and filamentous fungi, however, can be identified with very high probability to a species level [7, 8].

Except for taxonomical identification of bacteria, yeasts and filamentous fungi, MALDI-TOF MS is able to detect important resistance mechanisms (e.g., carbapenemases) and categorize microbes to susceptibility group based on their growth in presence of tested antibiotics or changes of protein profile after antibiotic exposure [9, 10, 11, 12]. Direct detection of microbes from clinical specimen is another challenge of current development in application of that technique in clinical microbiology [13, 14, 15]. As well as bacterial toxins can be efficiently detected by MALDI-TOF MS [16].

One of the main advantages of MALDI-TOF MS-based identification is the direct deposition of intact bacterial/yeast cells on the MALDI target. Then, cells are overlaid by a matrix solution. Thus, no further sample manipulation is needed. For better quality of spectra acquisition resulting in a higher score of species identification, specific extraction protocols can be used [7]. Simply, the spot target with the microbe can be overlaid by 1 microliter of 70% formic acid. In some microbes (e.g., yeasts and anaerobic bacteria), in tube extraction with ethanol and formic acid/acetonitrile may provide an advantage solution for spectra acquisition [7]. However, direct spotting, usually performed by a wooden toothpick, does not allow standardization of the amount of microbes deposited on the target spot. This process is highly dependent on the experience of technician and may significantly influence identification score.

Automation of colony picking for matrix-assisted laser desorption/ionization time-of-flight mass spectrometry (MALDI-TOF MS) identification and antibiotic susceptibility testing is still under development. One of the first devices enabling automatic deposition of colonies on the MALDI target is Copan Colibri (COPAN Diagnostics Inc., CA, USA), which was introduced during 25th ECCMID in 2015 in Copenhagen [17].

We describe here a novel technique for bacterial and yeast deposition on MALDI target using an automated workflow resulting in an increase of the score of microbe identification. We also provide a comparison of four different sample deposition methods.

## Materials and methods

### Bacterial isolates

The microbes were cultivated on 5% sheep blood agar, Endo agar, Candi Select agar (Bio-Rad, Prague, Czech Republic), PVX agar, or SCS agar (bioMérieux, Prague, Czech Republic) at 35°C overnight.

All samples were collected in routine diagnostic laboratory of University Hospital in Plzen (Czech Republic) which is a tertiary care hospital providing a complete medical service, including transplantology. Thus, community-acquired as well as hospital acquired pathogens are routinely identified in the laboratory. Mostly, clinical materials of upper and lower respiratory tract, gastrointestinal tract, urogenital tract, wounds and exudates, or blood cultures were included in the study.

### Methods for manual deposition of bacteria/yeasts on MALDI-TOF MS target

For direct spotting on the MALDI target (MSP 96 Target, Catalog No. 224989, Bruker Daltonics GmbH, Bremen, Germany), bacterial culture was transferred by a toothpick forming a thin film on the spot. After drying, the spot was covered by 1 microliter of a matrix solution [10 mg/ml of alfa-cyano-4-hydrocinnamic acid (CHCA) (Bruker Daltonik, Bremen, Germany) in 50% acetonitrile (Sigma-Aldrich, Prague Czech Republic) and 2.5% trifluoroacetic acid (Sigma-Aldrich, Prague Czech Republic)].

On-plate extraction (semi-extraction) was performed by spotting of bacteria as described above. After drying, the spot was covered by 1 microliter of 70% formic acid and allow to dry. Then, 1 microliter of matrix was applied.

In the wet deposition, 1 microliter of 70% formic acid was pipetted on the spot. Instantly, bacteria were collected manually with a platinum inoculation loop and resuspended, in the formic acid droplet, on the spot. After the spot was dried, 1 microliter of the matrix was applied.

### Automatic deposition using MALDI Colonyst

MALDI Colonyst (Biovendor Instruments, Brno, Czech Republic) was used for automatic sample preparation according to the manufacturer instructions. Shortly, three vials were loaded into the machine (70% formic acid, MALDI matrix solution and 70% acetonitrile as Washing solution respectively) and the system has been automatically washed prior spotting of bacteria. Additionally, MALDI target was inserted into the holder and automatic picking process has been started. Within each round, the Petri dish was inserted into the holder and colonies were selected by the simple mouse click within the high-resolution pictures in the MALDI Colonyst software tool and automatically picked by the platinum tips of the robot. Colony deposition was performed using “wet deposition” into the automatic pre-spotted formic acid droplet as described above. Colony picking and their deposition as well as MALDI matrix deposition were carried out automatically. The quality of deposition process has been automatically documented using screen shots of each step of the deposition process. The photos are automatically stored in the computer.

### MALDI-TOF MS measurement

Spectra acquisition was performed on MicroFlex LT mass spectrometer (Bruker Daltonik, Bremen, Germany) using MALDI Biotyper software version 3.1. Bruker Bacterial Test Standard was used for a calibration.

### Statistical analysis

To determine statistically significant differences, repeatability of automatic deposition method was determined. Thirty different microbial isolates (8 Gram-positive microbes, 8 Gram-negative microbes, 6 anaerobes, and 8 yeasts) were measured and processed by MALDI Colonyst robot. Each isolate was spotted ten times from the same culture plate. For each microbial group (Gram-negative bacteria, Gram-positive bacteria, anaerobe bacteria and yeasts), arithmetic mean of identification score (x), standard deviation (SD) and relative standard deviation (RSD%) were determined. Based on those parameters, the accuracy of the method and the reproducibility of the parallel measurements was evaluated [18].

## Results

An average relative standard deviation (RSD%) of automatic method was below 5.00% for each microbial group. Higher RSD% were identified in *Candida pararugosa* and *Peptostreptococcus micros* only (RSD% 8.21 and 5.56 respectively). Excellent repeatability was detected in Gram-negative bacteria (RSD% 1.81), followed by Gram-positive microbes. The lowest RSD were identified in yeasts and anaerobic bacteria (Table 1).

**Table 1:** Repeatibility of automatic bacterial deposition using MALDI Colonyst determined by standard deviation (SD) and relative standard deviation (RSD%). Each strain was spotted ten times from the same culture plate and identified using MALDI-TOF MS.

Species	$\dot{x}$	SD	RSD%	Species	$\dot{x}$	SD	RSD%	Species	$\dot{x}$	SD	RSD%	Species	$\dot{x}$	SD	RSD%
*K*. *pneumoniae*	2.39	0.03	1.21	*S*. *agalactiae*	2.41	0.06	2.33	*C*. *albicans*	2.13	0.07	3.27	*F*. *nucleatum*	2.21	0.04	1.69
*K*. *oxytoca*	2.26	0.05	2.19	*S*. *pyogenes*	2.30	0.08	3.66	*C*. *parapsilosis*	1.97	0.04	2.11	*F*. *magna*	2.16	0.05	2.25
*E*. *coli*	2.31	0.04	1.69	*S*. *pneumoniae*	2.25	0.06	2.89	*C*. *tropicalis*	2.04	0.06	3.01	*P*. *micros*	1.72	0.10	5.56
*P*. *aeruginosa*	2.48	0.07	2.67	*S*. *epidermidis*	2.17	0.04	1.81	*C*. *pararugosa*	1.79	0.15	8.21	*B*. *thetaiotaomicron*	2.35	0.06	2.51
*A*. *bereziniae*	2.27	0.05	2.13	*S*. *aureus*	2.49	0.04	1.41	*C*. *glabrata*	2.06	0.06	2.90	*C*. *innocuum*	2.02	0.03	1.70
*E*. *cloacae*	2.34	0.03	1.34	*E*. *faecalis*	2.44	0.04	1.77	*S*. *cerevisiae*	2.15	0.03	1.62	*C*. *difficile*	2.18	0.06	2.65
*P*. *mirabilis*	2.42	0.03	1.32	*S*. *warneri*	2.23	0.04	1.93	*C*. *lusitaniae*	2.27	0.05	2.17				
*C*. *freundii*	2.31	0.04	1.90	*C*. *amycolatum*	2.26	0.03	1.18	*C*. *guilliermondii*	2.07	0.09	4.18				
**Gram-negative microbe**	**2.35**	**0.04**	**1.81**	**Gram-positive microbe**	**2.32**	**0.05**	**2.12**	**Yeasts**	**2.06**	**0.07**	**3.34**	**Anaerobes**	**2.11**	**0.06**	**2.63**

In the first step of validation study 240 clinical isolates were used. The collection was composed of 100 Gram-positive bacteria, 100 Gram-negative bacteria, 20 anaerobes and 20 yeasts. In this step, identifications were performed by all four methods (direct spotting, semi-extraction, wet deposition and automatic deposition). All samples were measured in triplicates.

**Fig 1:**
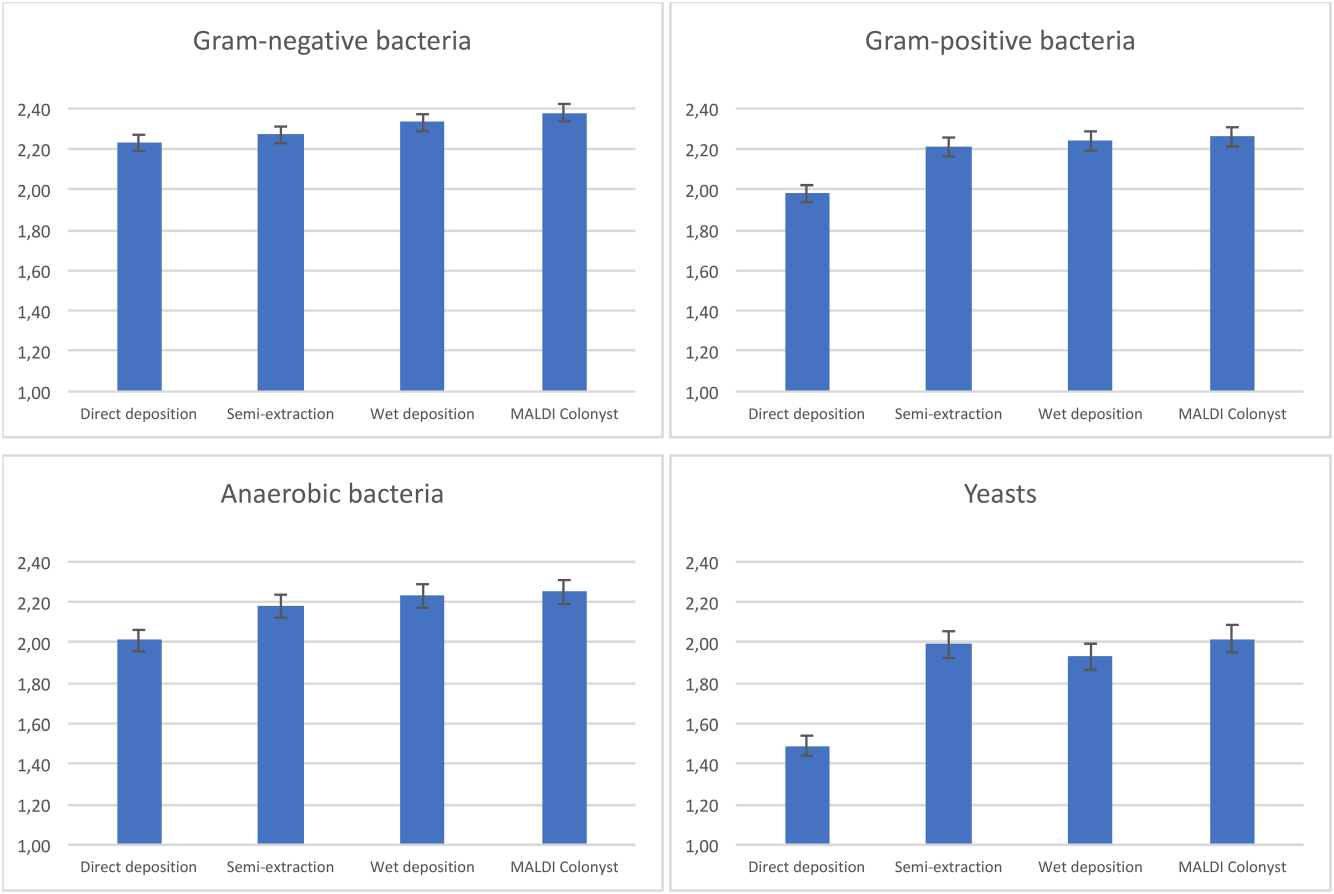
Comparison of different deposition methods with plotted RSD. Statistically significant differences are indicated by asterisks.

Table 2 shows an average identification score and error rate for each group of microbes and spotting methods. Average identification scores are calculated with and/or without errors (error is defined as an identification with a score below 1.70 or the spectra were no peaks were found). The spectra with no peaks found were excluded from the calculation of the average score. The lowest scores were obtained by manual toothpick spotting which significantly differ from other methods. Identification score of other methods did not significantly differ using calculated RSD. Nevertheless, the best results with low error rate have been observed using MALDI Colonyst robot (see Fig 1).

**Table 2:** Identification score of microbes processed by four different methods of deposition on MALDI target.

		Gram-positive	Gram-negative	Anaerobes	Yeasts
	Nr. of strains identified	100	100	20	20
	Total nr. of identifications	300	300	60	60
**Direct spotting**	Nr. of "score below 1.70"	46	5	14	40
	Nr. of "no peaks found"	3	1	0	7
	Nr. of total errors	49	6	14	47
	Total errors [%]	16.33%	2.00%	23.33%	78.33%
	Average identification score w/o errors*	2.09	2.24	2.17	1.87
	Average identification score with errors**	1.98	2.23	2.01	1.49
**Semi-extraction**	Nr. of "score below 1.70"	5	4	3	1
	Nr. of "no peaks found"	3	0	0	1
	Nr. of total errors	8	4	3	2
	Total errors [%]	2.67%	1.33%	5.00%	3.33%
	Average identification score w/o errors*	2.21	2.27	2.21	2.00
	Average identification score with errors**	2.21	2.27	2.18	1.99
**Manual wet deposition**	Nr. of "score below 1.70"	6	0	1	7
	Nr. of "no peaks found"	2	0	0	1
	Nr. of total errors	8	0	1	8
	Total errors [%]	2.67%	0.00%	1.67%	13.33%
	Average identification score w/o errors*	2.24	2.33	2.25	1.97
	Average identification score with errors**	2.24	2.33	2.23	1.93
**Automatic deposition**	Nr. of "score below 1.70"	5	0	1	1
	Nr. of "no peaks found"	2	0	0	0
	Nr. of total errors	7	0	1	1
	Total errors [%]	2.33%	0.00%	1.67%	1.67%
	Average identification score w/o errors*	2.28	2.38	2.26	2.03
	Average identification score with errors**	2.26	2.38	2.25	2.02

*/** Error is defined as the score with a value below 1.70, or spectra with no peaks (“no peaks found”). The spectra characterized as “no peaks found” were excluded from the calculation of the average identification score.

In general, the lowest error rate (no acquisition of spectra or no identification possible) was identified in Gram-negative bacteria in all tested groups. The highest error rate has been observed in yeasts deposited by a manual method using a toothpick (78.33%).

In the second step of the study, 542 clinical isolates were included. Those isolates consisted of 244 Gram-negative aerobic/facultative bacteria, 213 Gram-positive bacteria, 46 anaerobic bacteria and 39 yeasts. The isolates were simultaneously spotted by manual method (direct spotting for bacteria, semi-extraction for yeasts) and by automatic deposition using MALDI Colonyst. Colonies for both methods were selected from the same culture plate.

Automatic deposition showed better identification score in all groups (in Gram-positive, Gram-negative, anaerobes and yeasts)–see Table 3. The highest difference was observed in yeasts (2.02 and 2.14 respectively), anaerobes (2.04 and 2.22 respectively) and in some Gram-positive bacteria. On the other hand, the lowest difference in Gram-negative bacteria, where both methods differ by 0.07 only. All differences, however, were higher than the reproducibility of the method.

**Table 3:** Comparison of identification scores in the second stage of the study. Routine clinical samples were identified by direct spotting or semi-extraction (yeasts) and by automatic deposition using MALDI Colonyst from the same culture.

Bacteria	Nr. of isolates	Manual deposition average score	Deposition by Colonyst average score
**Gram-positive microbes (total number)**	213	2.20	2.31
***Staphylococcus* sp.**	71	2.17	2.25
***Streptococcus* sp.**	52	2.27	2.34
***Enterococcus* sp.**	63	2.25	2.39
**Other Gram-positive microbes**	27	2.01	2.18
**Gram-negative microbes (total number)**	244	2.30	2.37
**Enterobacteriaceae**	171	2.33	2.40
**Non-fermenting bacteria**	44	2.25	2.34
**Other Gram-negative microbes**	29	2.22	2.30
**Anaerobes**	46	2.04	2.22
**Yeasts**	39	2.02	2.14

## Discussion

The main focus of clinical microbiology is the isolation and identification of a pathogenic bacteria and subsequent determination of their susceptibility to antimicrobial agents. Currently, the MALDI-TOF MS method is the most common technique used for taxonomic identification in many states.

The aim of the study was to compare results of average identification score for routinely used manual deposition (semi-extraction for yeasts) with results of automatic deposition using MALDI Colonyst robot. Spotting by MALDI Colonyst significantly increased identification score of bacteria comparing with routine diagnostic process. The lowest difference was found in *Enterobacteriaceae* isolates which usually possess high identification score comparing with other microbes. Their cells can be easily disrupted during drying on the spot and after covering by matrix solution. Similar results were also observed in yeasts, because of the fact, that semi-extraction method is routinely used in those microbes. In this study, we used only commonly processed isolates in routine practice of our laboratory. Therefore, identification score and thus efficiency of automatic colony deposition may differ in laboratories with different patient spectra and isolated yeast’s species.

Results from routine testing were in concordance with the comparison of four spotting methods tested on 240 clinical isolates. Semi-extraction, wet deposition and automatic spotting using MALDI Colonyst provided similar identification score, significantly higher the scores obtained after direct deposition. No difference was observed in Gram-negative bacteria.

One of the main advantages of automatic deposition on a target is a semi-standardization of biomass amount in the spot. After picking up by the platinum loop, only a part of microbes is resuspended into the droplet of 70% formic acid. A part of biomass remains attached on the loop and is removed during sterilization process. During drying of the spot, the lyses is enhanced, and final spot is homogenously covered by bacterial lysate. Homogenous surface of the spot may also explain higher identification score in both wet-deposition techniques. Our results are in agreement with the publication of Theel *et al*. [19] demonstrating that direct deposition of *Corynebacterium* spp. to a formic acid drop may enhance identification score of these bacteria.

Another advantage of automatic colony deposition using MALDI Colonyst is the significant decrease of consumable cost, since there is no need of pipette tips for formic acid deposition (in case of semi-extraction) and matrix deposition. Based on standard MALDI preparation procedure, the tip should be replaced after each spot. Such a decrease of the running costs shall lead to increase of the number of selected colonies from each of the Petri dish for MALDI identification while not extending the preparation time and manual workload. Deposition of one 96 MALDI target takes approximately 45 minutes using MALDI Colonyst. This time includes loading plates into the instrument, selecting colonies from images on the screen, drying of the spot, pipetting and drying of the matrix. Manual deposition of one MALDI plate takes the similar time (ca. 40 minutes) dependent on the technician’s skills. The reported cost of the robot, however, is comparable with other automats used in clinical microbiology or biochemistry (catalogue price ca. 60 000 EURO). Thus, the initial investment to the machine may limit its use especially in small laboratories, because the cost of one spotting is therefore dependent on number of identifications processed daily. Last but not least, the whole automated deposition process is well monitored and recorder for further validation and retrospective quality control.

Described wet-deposition method allows integrating of MALDI-TOF mass spectrometry into automatic microbiology lines. Another possibility for automatic deposition of microbes on the MALDI target may be performed by pipetting of prepared inocula (e.g., for antibiotic susceptibility testing) on the spot. This procedure, however, may be more expensive, because not all microbes taxonomically identified in routine laboratory are subjected for antibiotic susceptibility testing and therefore, preparing of inocula is redundant.

## Supporting information

S1 File Validation of MALDI-Colonyst robot—Raw data. (XLSX)

S2 File Comparison of four different spotting methods—Raw data. (XLSX)

S3 File Determination of RSD—Raw data. (XLSX)

## References

[1] Croxatto A, Prod’hom G, Faverjon F, Rochais Y, Greub G. Laboratory automation in clinical bacteriology: what system to choose? Clinical Microbiology and Infection. 2016; 22: 217–235. doi: 10.1016/j.cmi.2015.09.030 26806135 10.1016/j.cmi.2015.09.030

[2] Ledeboer NA, Dallas SD. The Automated Clinical Microbiology Laboratory: Fact or Fantasy? Journal of Clinical Microbiology. 2014; 52: 3140–3146. doi: 10.1128/JCM.00686-14 24648549 10.1128/JCM.00686-14 PMC4313129

[3] Buchan BW, Ledeboer NA. Emerging technologies for the clinical microbiology laboratory. Clinical Microbiology Reviews. 2014; 27: 783–822. doi: 10.1128/CMR.00003-14 25278575 10.1128/CMR.00003-14 PMC4187641

[4] Lay JO. MALDI-TOF mass spectrometry of bacteria. Mass Spectrometry Reviews. 2001; 20: 172–194. doi: 10.1002/mas.10003 11835305 10.1002/mas.10003

[5] Patel R. MALDI-TOF MS for the diagnosis of infectious diseases. Clinical Chemistry 2015; 61: 100–111. doi: 10.1373/clinchem.2014.221770 25278500 10.1373/clinchem.2014.221770

[6] Fournier PE, Drancourt M, Colson P, Rolain JM, La Scola B, Raoult D. Modern clinical microbiology: new challenges and solutions. Nature Reviews Microbiology. 2013; 11: 574–85. 24020074 10.1038/nrmicro3068 PMC7097238

[7] Clark AE, Kaleta EJ, Arora A, Wolk DM. Matrix-assisted laser desorption ionization-time of flight mass spectrometry: a fundamental shift in the routine practice of clinical microbiology. Clinical Microbiology Reviews. 2013; 26: 547–603. doi: 10.1128/CMR.00072-12 23824373 10.1128/CMR.00072-12 PMC3719498

[8] Amlerová J, Studentová V, Hrabák J. Identification of *Mycobacterium* spp. isolates using matrix-assisted laser desorption/ionization-time-of-flight mass spectrometry (MALDI-TOF MS). Epidemiol Mikrobiol Imunol. 2014; 63: 196–199. 25412483

[9] Hrabák J, Chudácková E, Walková R. Matrix-assisted laser desorption ionization-time of flight (maldi-tof) mass spectrometry for detection of antibiotic resistance mechanisms: from research to routine diagnosis. Clin Microbiol Rev. 2013; 26: 103–114. doi: 10.1128/CMR.00058-12 23297261 10.1128/CMR.00058-12 PMC3553667

[10] Maxson T, Taylor-Howell CL, Minogue TD. Semi-quantitative MALDI-TOF for antimicrobial susceptibility testing in *Staphylococcus aureus*. PLoS One. 2017; 12: e0183899 doi: 10.1371/journal.pone.0183899 28859120 10.1371/journal.pone.0183899 PMC5578647

[11] Jung JS, Eberl T, Sparbier K, Lange C, Kostrzewa M, Schubert S, et al Rapid detection of antibiotic resistance based on mass spectrometry and stable isotopes. Eur J Clin Microbiol Infect. Dis. 2014; 33: 949–955. doi: 10.1007/s10096-013-2031-5 24338093 10.1007/s10096-013-2031-5

[12] De Carolis E, Vella A, Florio AR, Posteraro P, Perlin DS, Sanguinetti M, et al Use of matrix-assisted laser desorption ionization-time of flight mass spectrometry for caspofungin susceptibility testing of Candida and Aspergillus species. J Clin Microbiol. 2012; 50: 2479–2483. doi: 10.1128/JCM.00224-12 22535984 10.1128/JCM.00224-12 PMC3405623

[13] Arca-Suárez J, Galán-Sánchez F, Marin-Casanova P, Rodríguez-Iglesias MA. Direct identification of microorganisms from thioglycolate broth by MALDI-TOF MS. PLoS One. 2017; 12: e0185229 doi: 10.1371/journal.pone.0185229 28934331 10.1371/journal.pone.0185229 PMC5608331

[14] Faron ML, Buchan BW, Ledeboer NA. Matrix-Assisted Desorption Ionization Time of Flight Mass Spectrometry for the Use with Positive Blood Cultures: Methodology, Performance, and Optimization. J Clin Microbiol. 2017 10.1128/JCM.00868-17 PMC5703799 28855303

[15] Huang B, Zhang L, Zhang W, Liao K, Zhang S, Zhang Z, et al Direct Detection and Identification of Bacterial Pathogens from Urine with Optimized Specimen Processing and Enhanced Testing Algorithm. J Clin Microbiol. 2017; 55:1488–1495. doi: 10.1128/JCM.02549-16 28249997 10.1128/JCM.02549-16 PMC5405266

[16] Kalb SR, Boyer AE, Barr JR. Kalb SR, Boyer AE, Barr JR. Mass Spectrometric Detection of Bacterial Protein Toxins and Their Enzymatic Activity. Toxins (Basel). 2015; 7: 3497–511. 26404376 10.3390/toxins7093497 PMC4591662

[17] Dauwalder O, Landrieve L, Laurent F, de Montclos M, Vandenesch F, Lina G. Does bacteriology laboratory automation reduce time to results and increase quality management? Clinical Microbiology and Infection. 2016; 22: 236–243. doi: 10.1016/j.cmi.2015.10.037 26577142 10.1016/j.cmi.2015.10.037

[18] Bland JM, Altman DG. Statistics notes: Measurement error. British Medical Journal. 1996; 312: 1654.

[19] Theel ES, Schmitt BH, Hall L, Cunningham SA, Walchak RC, Patel R, et al Formic acid-based direct, on-plate testing of yeast and *Corynebacterium* species by Bruker Biotyper matrix-assisted laser desorption ionization-time of flight mass spectrometry. Journal of Clinical Microbiology. 2012; 50: 3093–3095. doi: 10.1128/JCM.01045-12 22760034 10.1128/JCM.01045-12 PMC3421773

